# Identification of novel transcripts and noncoding RNAs in bovine skin by deep next generation sequencing

**DOI:** 10.1186/1471-2164-14-789

**Published:** 2013-11-14

**Authors:** Rosemarie Weikard, Frieder Hadlich, Christa Kuehn

**Affiliations:** 1 Leibniz Institute for Farm Animal Biology (FBN), Institute of Genome Biology, Dummerstorf 18196, Germany

**Keywords:** Transcriptome, RNAseq, Skin, Pigmentation, Noncoding RNA, Novel transcripts, Cattle

## Abstract

**Background:**

Deep RNA sequencing (RNAseq) has opened a new horizon for understanding global gene expression. The functional annotation of non-model mammalian genomes including bovines is still poor compared to that of human and mouse. This particularly applies to tissues without direct significance for milk and meat production, like skin, in spite of its multifunctional relevance for the individual. Thus, applying an RNAseq approach, we performed a whole transcriptome analysis of pigmented and nonpigmented bovine skin to describe the comprehensive transcript catalogue of this tissue.

**Results:**

A total of 39,577 unique primary skin transcripts were mapped to the bovine reference genome assembly. The majority of the transcripts were mapped to known transcriptional units (65%). In addition to the reannotation of known genes, a substantial number (10,884) of unknown transcripts (UTs) were discovered, which had not previously been annotated. The classification of UTs was based on the prediction of their coding potential and comparative sequence analysis, subsequently followed by meticulous manual curation. The classification analysis and experimental validation of selected UTs confirmed that RNAseq data can be used to amend the annotation of known genes by providing evidence for additional exons, untranslated regions or splice variants, by approving genes predicted *in silico* and by identifying novel bovine loci. A large group of UTs (4,848) was predicted to potentially represent long noncoding RNA (lncRNA). Predominantly, potential lncRNAs mapped in intergenic chromosome regions (4,365) and therefore, were classified as potential intergenic lncRNA. Our analysis revealed that only about 6% of all UTs displayed interspecies conservation and discovered a variety of unknown transcripts without interspecies homology but specific expression in bovine skin.

**Conclusions:**

The results of our study demonstrate a complex transcript pattern for bovine skin and suggest a possible functional relevance of novel transcripts, including lncRNA, in the modulation of pigmentation processes. The results also indicate that the comprehensive identification and annotation of unknown transcripts from whole transcriptome analysis using RNAseq data remains a tremendous future challenge.

## Background

Advances in genome research such as next generation RNA sequencing technology (RNAseq) have opened a new horizon for the annotation of whole transcriptomes and understanding of global gene expression. The powerful RNAseq approach enables the unravelling of expression profiles underlying phenotypic, metabolic and physiological variability, as well as different developmental stages and environmental influences at single base resolution. A further advantage of this whole transcriptome sequencing technology is the ability to decipher unannotated transcriptional activity by identifying numerous novel transcripts (protein coding and noncoding) and additional alternative splice variants of known annotated transcripts [1-3].

The completeness of a comprehensive transcript catalogue for each species will depend on the collection of tissues, cell lines and phenotypes, as well as on the variety of physiological and developmental conditions, included. Compared to the well-investigated transcriptomes of humans and mice [4-9], the bovine transcriptomic repertoire is still far from being identified at a high resolution. Recently, several studies have evaluated whole transcriptome expression by applying RNAseq technology in specific bovine tissues or cells at distinct physiological, metabolic, developmental, disease or behavioural states; for example to describe transcriptome variation of milk and mammary gland gene composition [10,11], nutrient response [12,13], embryonic development and reproduction (e.g., [14-16]), stress response at weaning [17], negative energy balance at lactation [18], and phenotypic breed differences [19]. In these recent RNAseq studies in cattle, a variety of novel transcripts were detected, which had not previously been annotated in the bovine reference genome assembly. The hitherto existing studies provide evidence that deep RNA sequencing is a powerful approach to enumerate the comprehensive pool of various RNA classes associated with a defined phenotype.

With the aim to study pigmentation phenomena of bovine skin in the future, the focus of our present study was to describe the comprehensive transcript catalogue of this tissue. The key genes modulating mammalian pigment biosynthesis and melanocyte development including a variety of melanosomal components are well known [20-24]. Currently, 378 loci associated with coat colour have been identified in mice and their zebrafish and human homologues (according to the European Society for Pigment Cell Research, http://www.espcr.org/micemut/). There is also a wide variability in coat colour patterns within and between cattle breeds, which makes them suitable for studying function and regulation of loci affecting skin pigmentation. There is a variety of studies aiming at the identification of the molecular background of coat colour patterns in cattle (for overview see http://homepage.usask.ca/~schmutz/CowPatterns.html), but the specific causes of pigmentation variability, such as piebald spotting, coat colour dilution and coat disorders associated with pigmentation are still not completely clarified.

Using the whole transcriptome analysis approach of RNAseq on pigmented and nonpigmented areas of bovine skin, the specific focus of our study was to describe the comprehensive transcriptome of this tissue as well as to identify and annotate novel transcripts expressed in this tissue, including noncoding RNA (ncRNA).

During the recent decade the complex class of long noncoding RNA (lncRNA) has become increasingly recognised in the human and mouse transcriptomes and attracted significant attention in molecular research [4,8,25-27]. Numerous studies have provided evidence that lncRNA are an important component of regulatory architecture and mechanisms involved in chromatin modification, epigenetic regulation, genomic imprinting, transcriptional control as well as pre- and posttranslational mRNA processing. The functions of lncRNA are reported to be associated with pathogenesis of many diseases (e.g., tumour growth, mental and psychiatric disorders) and developmental and differentiation processes [25,28-34]. However, the biological function and significance of lncRNA is still the subject of intense debate [26,35,36]. Recent studies revealed that there are still many novel lncRNAs to be detected for the well-analysed transcriptomes like human and mouse [9,37,38]. While our knowledge of ncRNAs has been expanding thanks to the identification and annotation of diverse classes of ncRNAs from human, mouse and other species, currently, there is very limited knowledge available about ncRNA distribution and function in farm animal genomes. Very recently, a regulatory function for a bovine long intergenic ncRNA (lincRNA) in horn bud differentiation in *Bovidae* had been suggested [39]. Tissue-specific, ectopic overexpression of this lincRNA was found to be the most plausible cause of horn bud agenesis and the bovine polled phenotype.

We postulate that transcripts not yet annotated in the bovine genome assembly, like ncRNA, may play a regulatory role for the expression of complex pigmentation phenotypes and epidermal processes of bovine skin. Therefore, we used a deep RNAseq approach to elucidate the complex pool of unknown transcripts and ncRNAs yielded from divergently pigmented bovine skin to identify novel transcripts and ncRNAs expressed in this tissue. Novel gene information from bovine skin cell transcriptomes can be used for further gene expression studies in skin tissue, to contribute to the elucidation and molecular understanding of pigmentation processes and to gain a deeper functional annotation of the bovine genome and transcriptome.

## Methods

### Phenotypes and sampling

Two skin samples differing in their pigmentation pattern (pigmented and nonpigmented) were collected from each of two bulls with a piebald phenotype. The bulls were from a F2 resource population generated from a cross of Charolais × German Holstein [40]. The bulls were homozygous for a dominant black mutation p. Tyr155ter in the *MC1R* (melanocortin 1 receptor) gene (extension locus) [20] and heterozygous for the dilute mutation c.64G > A in the *PMEL* (premelanosome protein) gene [41]. Differentially pigmented skin samples from closely adjacent skin areas were taken at slaughter at 18 months of age, trimmed from fat tissue, cut in small pieces, snap-frozen in liquid nitrogen and stored at −70°C until further processing.

### Library preparation and sequencing

Total skin RNA was extracted as has been described [42] using the NucleoSpin RNA II kit (Macherey & Nagel, Düren, Germany). A digestion step with proteinase K was included after tissue lysis and grinding using the Precellys 24 tissue homogenizer (peQLab, Erlangen, Germany). Genomic DNA was carefully eliminated from RNA preparations by repeated on-column digestion using twice the concentration of RNAse-free DNase I the manufacturers recommended in their protocols (Macherey & Nagel, Düren, Germany). Quality control of RNA was checked by a PCR specifically designed to detect genomic contamination [43]. RNA concentration and purity were measured using a NanoDrop ND-1000 spectrophotometer (peQLab, Erlangen, Germany). RNA integrity was assessed according to the intensity and shape of 28S and 18S rRNA bands by agarose gel electrophoresis and analysis on the Bioanalyzer 2100 (Agilent Technologies, Böblingen, Germany). High-quality RNA was used for mRNA library preparation using the Truseq RNA sample prep kit (Illumina, San Diego, CA) according to the manufacturer’s instructions (applying appropriate indices for multiplexing during cluster generation and sequencing).

The four individual RNAseq libraries were monitored for insert size using the Bioanalyzer 2100 (Agilent Technologies, Santa Clara, CA) and validated regarding sequence content by cloning an aliquot of each library into a plasmid vector (Zero Blunt TOPO PCR cloning kit, Invitrogen, Darmstadt; Germany) followed by sequencing of 40 randomly selected clones from each sublibrary.

Finally, a paired-end sequencing run with 2 × 61 cycles was performed on an Illumina GA IIx sequencing platform (Illumina, San Diego, USA). To this end, the four individual, indexed RNAseq libraries were pooled and aliquots were distributed across six lanes of the flow cell. Sequence reads were subjected to demultiplexing using the CASAVA 1.8 software (Illumina, San Diego, CA) followed by quality checking using the FastQC algorithm (http://www.bioinformatics.babraham.ac.uk/projects/fastqc/). FastQ files from individual lanes were merged for each sample and served as input for the following analyses.

### Reannotation, mapping and bioinformatic data analysis

Read alignment to the reference genome was performed using the Bowtie/ TopHat/ Cufflinks/ Cuffmerge pipeline [44]. A filtering step using SAMtools and Linux commands [45] was performed to eliminate those reads showing more than two mismatches to the reference genome and reads with multiple mapping hits. A guided transcript assembly using the bovine reference genome assembly UMD3.1 (ftp://ftp.ncbi.nlm.nih.gov/genomes/Bos_taurus/, downloaded 28/02/2012) on top of the Ensembl reference annotation, release 66, (ftp://ftp.ensembl.org/pub/release-66/gtf/bos_taurus/, downloaded 28/02/2012) was carried out for each sample file separately. This strategy considered the reference genome annotation and additionally, allowed inclusion of sequence reads mapping to chromosome regions or transcription units not yet annotated in the underlying reference transcript assembly. The separate analysis of the individual transcript assembly for each sample enabled the identification of potential differently spliced transcripts of pigmented and nonpigmented phenotypes. Thus, the generated final transcriptome assembly comprising transcripts from both phenotypes will provide novel transcripts, genes and isoforms in addition to the reannotated known reference loci.

Finally, the resulting individual transcript assemblies were merged to form a single transcript assembly using the Cuffmerge option. The merged transcript assembly (final GTF file) was applied for locus and transcript quantification using Cuffdiff v1.3. The final dataset represents the joint transcriptome of pigmented and nonpigmented skin samples including all transcripts (annotated and nonannotated) that contain at least one exon and reveal expression either in pigmented or nonpigmented skin samples. A further filtering step was included to eliminate transcripts having a very low expression level. All transcripts which had a lower bound of zero for the 95% confidence interval on the FPKM (fragments per kb for a million reads) of the object were excluded from the dataset. Transcript and locus assemblies were visualised by inspection of the BAM files of the samples and the final annotation with the IGV viewer [46].

### Analysis and classification of unknown transcripts

For the analysis and classification of unknown transcripts (transcripts with class code u according to Cufflinks), the entire transcript dataset (containing all transcripts previously annotated or nonannotated in the Ensembl reference assembly, release 66) was compared to the NCBI iGenome annotation (http://cufflinks.cbcb.umd.edu./igenomes.html, downloaded 03/11/2012) for *Bos taurus* using the Cuffcompare option to identify transcripts predicted in the NCBI database (*Bos taurus* UMD3.1). Predicted bovine NCBI transcripts are generally derived by automated computational analysis using the NCBI gene prediction method GNOMON (http://www.ncbi.nlm.nih.gov/genome/guide/gnomon.shtml). They are fully or partially supported by protein sequence records from several model organisms (XM-, XR- accession numbers). These predicted loci were not annotated in the Ensembl reference assembly, release 66. Subsequently, those transcripts initially classified as nonannotated in our dataset, but which corresponded to the predicted NCBI loci were eliminated from our data subset containing the nonannotated transcripts. This reduced dataset very conservatively represents the transcripts not previously annotated in the bovine transcriptome and served as final input for the following analyses of unknown transcripts (UTs) in our study on bovine skin.

### Comparative sequence analysis

For the characterisation of transcripts not yet annotated in the bovine genome assembly, sequence homology searches with UT sequences using BLASTN (v2.2.26+, e-value = 1e-11) were carried out in several different, publicly available RNA databases as summarised in Table 1. Furthermore, the dataset of UTs from bovine skin was compared with datasets of recently published putative bovine noncoding sequences [47,48]. The stringency criteria for sequence similarity were defined with a mapping identity of ≥75% and a total sequence identity of ≥90% in a covered region ≥100 nt for interspecies searches and with ≥90% of total sequence identity and mapping identity in a covered region ≥100 nt for intraspecies searches.

**Table 1:** Databases screened for sequence similarity

**Database**	**RNA class**	**Source/URL**	**Reference**
Rfam v11	RNA families	http://www.sanger.ac.uk/resources/databases/rfam.html	[49]
Refseq NCBI (15/12/2012)	Protein coding and noncoding RNA	http://blast.ncbi.nlm.nih.gov/Blast.cgi?	
Gencode v13	Protein coding and noncoding RNA	http://www.gencodegenes.org/	[9,50]
LNCipedia v1.2	Annotated human long noncoding RNA	http://www.lncipedia.org/	[51]
Noncode v3.0	Integrative annotation of noncoding RNA	http://www.noncode.org/	[52]
lincRNA (01/11/2012)	Annotated human long intergenic noncoding RNA	http://genome.ucsc.edu/cgi-bin/hgTrackUi?db=hg19&g=lincRNAs	[38]
RNAdb v2.0	Noncoding RNA	http://jsm-research.imb.uq.edu.au/rnadb/	[53]
lncRNA (01/11/2012)	lncRNAs in eukaryotes	http://lncrnadb.com	[54]
Dataset 1	Bovine noncoding RNA	Personal information of the authors	[47]
Dataset 2	Bovine long noncoding RNA	Supplemental information	[48]

The results were manually curated by reverse screening of the sequences with significant similarity hits against the NCBI database using BLAST tools. For this purpose, the sequence similarity searches in the NCBI nucleotide database were performed using MEGABLAST for highly similar sequences (within species: *Bos taurus* build 6.1), and BLASTN for somewhat similar sequences (interspecies searches: *Homo sapiens* annotation release 104, *Mus musculus* build 38.1, *Ovis aries* annotation release 100) applying default parameters. Interspecies sequence similarity was only accepted if mapping of the specific unknown bovine transcript and the sequence underlying the respective similarity hit indicated an orthologous chromosome area syntenic between both species. Sequence similarity within species was accepted if mapping results of the sequence underlying the respective similarity hit and the unknown bovine transcript were concordant and displayed identical adjacent loci. For manual curation of sequence similarity hits, a more restricted threshold filter for sequence similarity was defined in a covered region ≥150 nt with ≥75% identity for interspecies searches and with ≥95% sequence identity for intraspecies searches. If the interspecies sequence similarity was ≥90% but covered a shorter region, the initial sequence similarity hit was also accepted.

### Evaluation of coding potential

The prediction of a coding potential of transcripts not yet annotated in the bovine genome assembly was performed using the Coding Potential Calculator (CPC) algorithm (http://cpc.cbi.pku.edu.cn/), which is based on a support vector machine (SVM) [55]. We applied CPC (version 0.9-r2) using the complete UniRef90 database (http://www.ebi.ac.uk/uniprot/database/download.html, downloaded 22/06/2012). A positive CPC-score S indicates a protein coding potential of the respective target transcript, whereas negative CPC-S values predict noncoding potential of transcripts [55]. In general, the more the CPC-score differs from zero, the more reliable is the prediction by the CPC algorithm. To receive a higher reliability for the coding potential prediction, we set the threshold for reliable protein coding capacity at CPC-S ≥1, and UTs with a CPC-S ≤ −0.5 were predicted to be potentially noncoding. The UTs with a CPC-S between these limits were indexed as neutral with ambiguous coding potential.

In addition, an alignment-free algorithm, the Coding Potential Assessment Tool (CPAT) [56], http://lilab.research.bcm.edu/cpat) was applied (version 1.2.1) on our UT dataset in order to assess the coding potential of nonannotated transcripts by a second independent prediction method. Due to the limited annotation data available for *Bos taurus*, human reference RNA sequences (downloaded from NCBI) were applied as input source. According to the authors [56], the CPAT coding probability score ranges between 0 and 1, and the optimum cut-off for protein coding probability varies depending on the species to be analysed. The cut-off was determined to be in a range from 0.364 to 0.44 for human, zebrafish, fly and mouse. For reliable prediction of coding capacity of bovine UTs from our dataset, we chose a more conservative coding probability cut-off at ≥0.5 to extract putative protein coding sequences. In order to extract potential noncoding transcripts with a high reliability from our dataset, we selected a very stringent threshold for the CPAT probability and assigned UTs with a score <0.02 as ncRNA. The UTs with a score between the selected thresholds were classified to possess an ambiguous coding potential.

### Validation by RT-PCR

Transcript-specific primers for structural validation and tissue-specific expression analysis of the selected transcripts using RT-PCR were designed using OLIGO Primer Analysis Software (MedProbe, Oslo, Norway). The specificity of RT-PCR primers was checked by BLAST search against the *Bos taurus* reference transcriptome and genome assembly using the Primer-BLAST tool (http://www.ncbi.nlm.nih.gov/tools/primer-blast/index.cgi?LINK_LOC = BlastHome).

In addition to differentially pigmented skin, total RNA was extracted from seven additional bovine tissues (thyroid gland, adrenal gland, liver, lung, brain, mammary gland, skeletal muscle) collected from an adult individual of the Charolais × German Holstein F2 resource population [40]. Total RNA was isolated using the NucleoSpin RNA II kit (Macherey & Nagel, Düren, Germany). Quality check of the RNA was performed as described for the RNAseq library preparation. Only RNA samples without detectable DNA contamination were used for further processing in locus-specific RT-PCR experiments. The cDNA was synthesised by reverse transcription from 500 ng total RNA utilising the SuperScript First-Strand Synthesis System III for RT-PCR (Invitrogen, Darmstadt, Germany) according to the manufacturer’s instructions and applying a combination of 50 ng random hexamer and 50 pmol oligo (dT)_20_ primers. The cDNA reaction was performed in duplicate, purified using the NucleoSpin Extract II kit (Macherey & Nagel, Düren, Germany) and pooled. The purified cDNA pool was finally diluted with one volume of DNase/RNase-free water. After subsequent PCR, amplified cDNA fragments were purified using the NucleoSpin Extract kit II (Macherey & Nagel, Düren, Germany) and verified by sequencing. Sequences of transcript-specific primers used for transcript validation and tissue-specific expression analysis are given in Additional file 1. Sequences for primers of the reference genes (*GAPDH* and *EIF3K*) were used according to [57].

## Results and discussion

### Mapping and reannotation of the transcripts identified in bovine skin

Results of our RNAseq analysis in bovine skin demonstrate clearly that pervasive transcription also takes place in cattle tissues. This is in line with transcriptome-wide studies in human, mouse and other species have also discovered unprecedented high numbers of novel transcripts, a large fraction of which were ncRNAs (e.g., [4,5,7,25,50]). After demultiplexing, merging and filtering of reads, 38.2 – 75.2 million uniquely mapped fragments were obtained per skin sample in our experiment. A total of 25.8 Gbp were sequenced and successfully mapped to the reference genome assembly. Finally, 39,577 unique primary skin transcripts were assigned to the bovine genome. The majority of unique transcripts (65%) were mapped to reference gene regions annotated in the bovine reference genome assembly. 35.6% of the reference-mapped transcripts showed identity to known reference transcripts, and 29.4% of the transcripts were assigned to known transcript regions (including intronic regions), presumably displaying potentially novel isoforms for the respective transcripts. A total of 13,086 transcripts were found to be not annotated in the bovine genome assembly (33.1%). 2,202 of these transcripts could be assigned to genes that have been predicted in the bovine genome NCBI assembly by computational algorithms.

Investigation of the bovine skin transcripts, which already possessed a clear annotation in the bovine genome assembly, was not in the focus of this study. Our special emphasis was to identify and classify unknown transcripts, that is, those transcripts expressed in bovine skin but not yet annotated in the bovine genome assembly. We hypothesised that transcripts not yet annotated in the bovine genome assembly, like lncRNA, may play a regulatory role in the expression of phenotypes and disorders associated to pigmentation of bovine skin.

After cleansing the data set of 13,086 transcripts not annotated in the bovine genome assembly (corresponding to those transcripts with Cufflinks class code u, unknown intergenic transcripts) by subtracting transcripts with a gene prediction status in the NCBI Refseq database, the final dataset of unknown transcripts resulted in a total of 10,884 transcripts not yet annotated. These UTs represent transcripts mapping outside of known and predicted loci.

**Figure 1:**
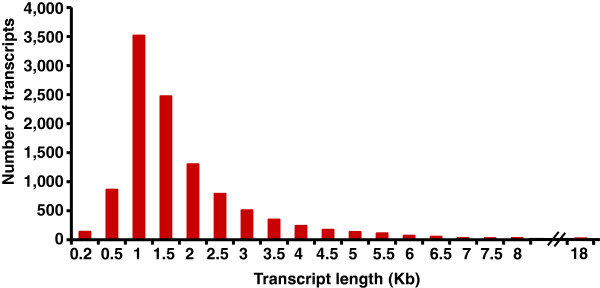
Length variation and number of unknown transcripts detected in bovine skin.

The size of the UTs mapped in the bovine genome assembly ranged from 62 to 17,500 bp. Most of them had a size varying between 500 bp and 2 kb (Figure 1). The UTs consisted of single or multiple exons (up to 10). However, the majority of them (91%) showed a bias toward single exon structure (9,974 transcripts).

**Figure 2:**
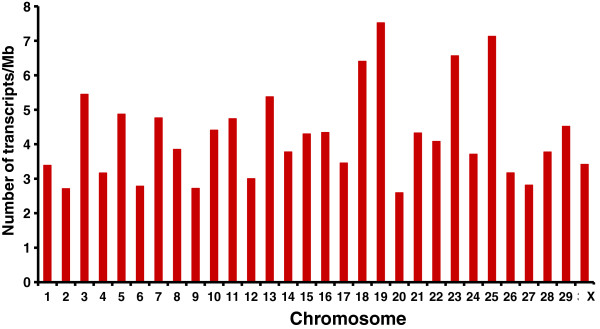
Number of unknown transcripts detected in bovine skin per chromosome.

**Figure 3:**
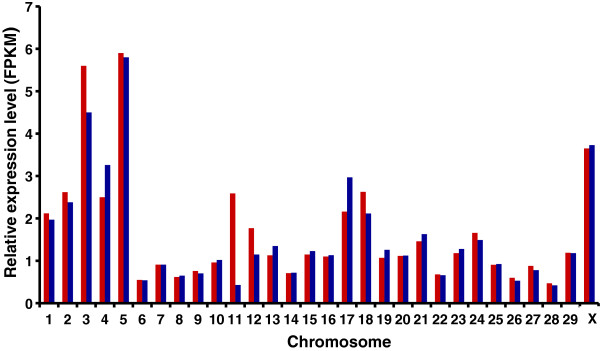
**Average relative expression level of unknown transcripts in pigmented and nonpigmented skin per chromosome.** Red: pigmented skin blue: nonpigmented skin FPKM: fragments per kb per transcript per million mapped reads.

The UTs were found to be not equally distributed on bovine chromosomes (Figure 2). The highest numbers of UTs were detected on bovine chromosomes (BTA) 18, 19, 23 and 25. Wide-spread transcription along all chromosomes with some bias in transcriptional activity on specific chromosomes indicates that the presence of UTs is not due to transcriptional noise. The highest average expression levels of UTs with a size >105 bp were observed on BTA3 and BTA5 (Figure 3). Interestingly, on these two chromosomes, several loci with functional relevance for epidermal and keratinocyte differentiation processes, skin disorders and pigmentation-associated processes are clustered. For example, the keratin type II gene cluster, *PMEL* (premelanosome protein), *BLOC1S1* (biogenesis of lysosomal organelles complex-1, subunit 1), *KITLG* (KIT ligand), and *ADAMTS20* (adam metallopeptidase with thrombospondin type 1 motif 20) are located on BTA5. On BTA3, the *LCE* (late cornified envelope) gene cluster, *KPRP* (keratinocyte proline-rich protein), *CRNN* (cornulin), *FLG* (filaggrin), *RPTN* (repetin), *TCHH* (trichohyalin, *IVL* (involucrin), *LOR*, (loricrin), and *MLPH* (melanophilin) are annotated. Differences in average expression levels of UTs between pigmented and nonpigmented skin were found on BTA11, 3, 12, 17 and 18. The substantial difference observed on BTA11 is mainly due to short unknown transcripts. The difference displayed on BTA3 is caused by several UTs mapped within the chromosomal region, where the *LCE* gene cluster is located, which is not completely annotated in the bovine genome compared to the human genome.

**Figure 4:**
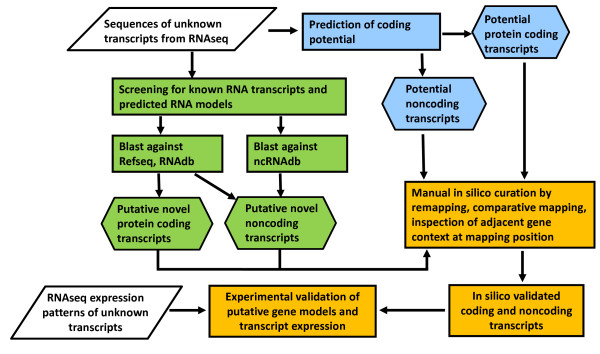
**Work flow for classification of unknown transcripts.** Refseq RNAdb: diverse RNA databases containing known coding RNA sequences. ncRNAdb: diverse RNA databases containing known noncoding RNA sequences. Rhomboid describes input data rectangle describes a process, hexagon describes different transcript categories.

Further characterisation and classification of the UTs dataset was performed according to the analysis work flow shown in Figure 4.

### Classification of unknown transcripts according to their protein coding prediction potential

The majority of ncRNAs detected in recent transcriptome studies were long noncoding RNA (lncRNAs). LncRNAs are defined as having a size >200 nt [58], but it has also been reported that lncRNAs lack discernible common features or structural motif facilitating categorisation and functional prediction (e.g., [38,59-61]). Many lncRNAs resemble protein coding RNAs; they are often capped, spliced and polyadenylated [62,63]. The challenging problem for identification of lncRNA is that the current coding potential prediction methods only work well for protein coding RNA. Therefore, the most widely used strategy to annotate a potential ncRNA is to exclude that the respective candidate ncRNA possesses protein coding features [64].

To predict the protein coding potential of UTs in our dataset, we applied two distinctive algorithms scanning for different lines of evidence for protein coding capacity, the Coding Potential Calculator (CPC, [55]) and the Coding Potential Assessment Tool (CPAT, [56]). Whereas CPC uses machine-learning based methods for modelling and extracting sequence and comparative genomics features, CPAT is an alignment-independent tool, which uses logistic regression to distinguish between coding and noncoding transcripts on the basis of four different sequence features. CPAT has been shown to have a higher performance in specificity, particularly in identifying ncRNA [56]. The analysis of the coding potential applying the CPC and CPAT tools on our dataset revealed similar results (Table 2). Both coding potential prediction tools assigned the majority of UTs to the ncRNA class (CPC: 62.5%, CPAT: 63.3%, Table 2). A high, putative coding potential was calculated for 6.8% and 2.3% of UTs by CPC and CPAT, respectively. For the remaining UTs the prediction could not be unequivocally made (CPC: 30.7%, CPAT: 34.4%, Table 2).

**Table 2:** Prediction of coding potential of unknown transcripts detected in bovine skin

**Prediction tool**	**Coding**	**Noncoding**	**Ambiguous**	**Inconsistent**
CPC	741	6,804	3,339	-
CPAT	251	6,889	3,744	-
Intersection (CPC + CPAT)	118	4,948	1,552	4,266

*CPAT*: Coding Potential Assessment Tool [56]*CPC*: Coding Potential Calculator [55].

For increased reliability of final classification, our dataset of 10,884 UTs was screened for candidate transcripts with an identical prediction by both coding potential prediction tools, CPAT and CPC. A total of 6,618 UTs (60.8%) showed a concordant classification (Table 2). The intersection between both prediction tools revealed 118 potentially protein coding transcripts (1.1%, Additional file 2) and 4,948 potentially ncRNA (45.5%, Additional file 3). A total of 1,552 transcripts (14%) could not be clearly classified regarding their coding potential based on the selected reliability thresholds by both tools. The remaining 4,266 transcripts (39%) were inconsistently categorised by both coding potential prediction tools. Notably, the majority of the 4,948 putative noncoding transcripts had a length >200 bp (4,849) and could therefore be designated as potential lncRNA (Additional file 3). These 4,849 potential lncRNAs represent a dataset that would be the most appropriate for laboratory follow-up studies.

### Characterisation of putative coding and noncoding transcripts by sequence similarity analysis

Currently, there is no catalogue of bovine ncRNAs from different cells and tissues available as there is for humans and mice. At the beginning of our RNAseq project, the lncRNA database reported a collection of eight bovine lncRNAs [54]. In the meantime, there were two reports aiming to identify bovine ncRNA. Both studies used the bovine Expressed Sequence Tag (EST) resources available from public databases, although the EST datasets were initially generated to identify and annotate novel protein coding genes. In the first study [47], 23,060 deposited ESTs were predicted and annotated as putative ncRNAs or ncRNA precursors by computational analysis in a genome-wide scale. The second study [48] used more stringent criteria and identified 449 putative bovine lncRNAs with at least two exons located in 405 intergenic regions (at least 1 kb away from known genes). Searching for sequence similarity of the 10,884 bovine skin transcripts from our UTs dataset with putative bovine ncRNA sequences from these two EST-based datasets [47,48] did not yield any identical sequences. This may be due to the fact that ESTs unique to skin tissue were underrepresented in the *Bos taurus* EST resources. Cell/tissue and time-specific expression of lncRNA has been reported for other species [38,50,65], a feature defined as a specific characteristic of this RNA class and prerequisite for their function in gene expression regulation. These temporally and spatially restricted expression patterns, together with their relatively low expression levels, may explain why our skin lncRNAs showed no overlap with noncoding transcripts of the two previous reports. Thus, comprehensive sampling and study of tissues and developmental stages is required to discover a complete lncRNA set of a species’ genome.

Usually, lncRNAs were found to be more plastic than protein coding genes, to evolve more rapidly and to display no stringent interspecies sequence conservation analogous to protein coding RNA [60,66]. This is a challenging problem for the identification of lncRNA by comparative sequence analysis. However, there are also reports about a small population of conserved lncRNA sequences displaying a moderate degree of sequence similarity or similar specific sequence elements across mammalian species, for which a potential function is assumed [34,38,67,68]. Qu and Adelson [34] concluded from their comprehensive evaluation of available lncRNA-related studies across species that lncRNAs are less conserved than protein coding genes but still exhibit a clear conservation compared to non-functional genomic elements.

To classify UTs of our dataset and to identify lncRNAs conserved in other species, sequence homology searches for known and predicted transcripts were carried out (Table 1). A summary of the results is presented in Table 3 indicating that 688 out of the 10,884 UTs (6.3%) from our dataset displayed conserved interspecies sequence similarity. Detailed information on sequence similarity of the 688 transcripts obviously conserved between species is provided in Additional file 4.

**Table 3:** Sequence similarity of unknown transcripts to sequences detected in non-bovine RNA databases

**Category**	**UTs Similarity**	**Analysed databases**
lncRNA (including conserved lncRNA)	281 (227)	Gencode v13, Noncode v3.0, Lncipedia v1.2, NCBI refseq
Amended gene	67	NCBI refseq, Gencode v13
UTR of known gene	152	NCBI refseq, Gencode v13
Potential novel gene	46	NCBI refseq, Gencode v13
Pseudogene	96	NCBI refseq, Gencode v13, Noncode v3.0
Potential pseudogene	46	NCBI refseq, Gencode v13, Noncode v3.0

Screened databases were described in Table 1. Transcripts were categorised after manual curation of the similarity hits received from database searches.

Based on interspecies sequence similarity and conserved gene structure hypothesis, 219 UTs (2%) suggest the existence of additional exons or untranslated regions for bovine genes that are possibly incompletely annotated in the current bovine genome assembly (Table 3, Additional file 4). Furthermore, 46 UTs (0.4%) may represent potential novel bovine gene loci not yet annotated, 35 of which are supported by evidence from *ab initio* bovine gene models predicted by the GNOMON algorithm as well as by concordance with the structural organisation of the respective human orthologous genes. The results of sequence similarity search (Table 3) revealed that 281 UTs (2.6%) showed sequence similarity to human genome sequences that are located between annotated genes (Additional file 4). These transcripts may represent a particularly reliable primary dataset of putative bovine skin lncRNAs for subsequent detailed functional experiments. The majority of them (227, 80.8%) displayed conserved sequence similarity to known human and murine lncRNAs deposited in public RNA databases (Table 3). In addition, 96 UTs (0.9%) could be assigned to known pseudogenes, whereas 46 UTs (0.4%) could be predicted as potential pseudogenes (Table 3, Additional file 4). Potential pseudogene prediction was inferred from sequence similarity to a known human coding gene on one side but on the other side, the mapping position of the respective unknown bovine transcript in the bovine genome assembly was not conserved with that of the corresponding human gene (different adjacent gene context). In total, the 281 transcripts displaying interspecies sequence similarity and the 142 transcripts with pseudogene-characteristic assignments represent noncoding transcripts supported by conserved interspecies sequence information. Consequently, the remaining UTs predicted to possess noncoding potential should represent putative bovine-specific lncRNAs.

Out of the UTs with interspecies sequence similarity, 43 were predicted concordantly by CPC and CPAT to possess coding potential (see Additional file 2), whereas for 257 UTs, noncoding potential was assigned (see Additional file 3).

### Classification of unknown transcripts in relation to annotated genes

**Figure 5:**
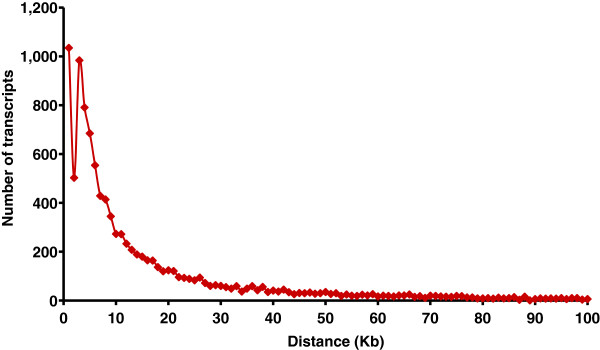
Distance of unknown transcripts their nearest neighbouring Refseq gene.

We further analysed the UTs with respect to their neighbouring protein coding genes to determine a potential transcriptional overlap with known bovine RefSeq genes. The alignment of the 10,884 UTs on the bovine reference genome assembly indicated that the transcripts not yet annotated were predominantly mapped in intergenic chromosomal regions (Figure 5). However, nearly 10% of the UTs (1,035) were found to be located within a 1 kb distance to an annotated locus (upstream and/or downstream). A sharp decline of the transcript frequency is displayed at a distance >3 kb (Figure 5).

For each putative protein coding and noncoding transcript, the closest flanking locus was identified and defined as the nearest neighbour (reference) locus (Additional files 2 and 3). The transcripts located within a distance ≤1 kb to a closely annotated adjacent locus were predicted to be likely related to UTRs of the respective nearest neighbour locus. Results from comparative similarity searches in diverse ncRNA databases revealed that a substantial number of identified sequence similarities were detected in regions close to known human and mouse genes (Additional file 4). This can possibly indicate missing UTRs of the respective orthologous bovine genes due to their incomplete annotation. However, it cannot be excluded that these UTs might belong to the classes of UTR-associated or UTR-related RNAs that have been discussed in the literature [27,47].

After excluding those transcripts located at a distance ≤1 kb from an annotated gene from the dataset containing the 4,848 putative lncRNAs, 4,365 of them (90%) remained in the dataset. Due to their intergenic position, these lncRNAs could be categorised as putative lincRNA. About 75% of putative lincRNAs had no neighbouring annotated gene within a distance of 5 kb.

### Validation of putative protein coding transcripts by sequence similarity analysis

To validate the results from the coding potential prediction analyses, the 118 potential protein coding transcripts (concordantly predicted by both coding potential prediction tools) were inspected manually by sequence similarity analysis. Comparative sequence alignments using BLAST tools on the bovine and human genome NCBI genome annotations (accession date 16/04/2013) revealed 62 putative protein coding transcripts (Additional file 2) supported by *ab initio* bovine models (predicted by the NCBI eukaryotic gene prediction tool, GNOMON and 20 pseudogene transcripts. Supported by the gene structure of human orthologs, 20 of the 62 putative protein coding transcripts identified bovine genes that obviously have been incompletely annotated in the bovine genome assembly, whereas 15 of them represent potential novel bovine transcript loci, which are structurally supported by the respective human orthologous genes. Out of the 36 remaining putative protein coding transcripts not supported by GNOMON gene prediction models, 32 were found to be located adjacently to loci annotated in the bovine genome. Only four putative coding transcripts were detected in intergenic regions.

In summary, for the majority (69%) of putative coding transcripts, a respective unambiguous syntenic chromosomal region could be mapped in the human genome assembly indicating a high degree of conservation of transcriptional activity. Consequently, these transcripts designated as putative protein coding could be excluded from the noncoding transcripts with a high reliability. However, their structure and expression have to be confirmed by further experimental validation.

Based on the results of manual curation of coding potential prediction for UTs by interspecies sequence similarity comparison, we conclude that a consistent assignment of an unknown transcript by both bioinformatic coding potential prediction tools might assist the identification of putative noncoding transcripts. However, the results clearly showed that it still remains difficult to reliably distinguish lncRNAs from protein coding mRNAs in a huge dataset of unannotated transcripts based only on excluding transcripts with potential functional coding capacity. Prediction accuracy of computational prediction algorithms aiming at specific identification of lncRNAs has to be improved, but depends on qualified training datasets for lncRNA classification in the targeted species.

### Experimental validation of selected putative coding and noncoding transcripts

**Figure 6:**
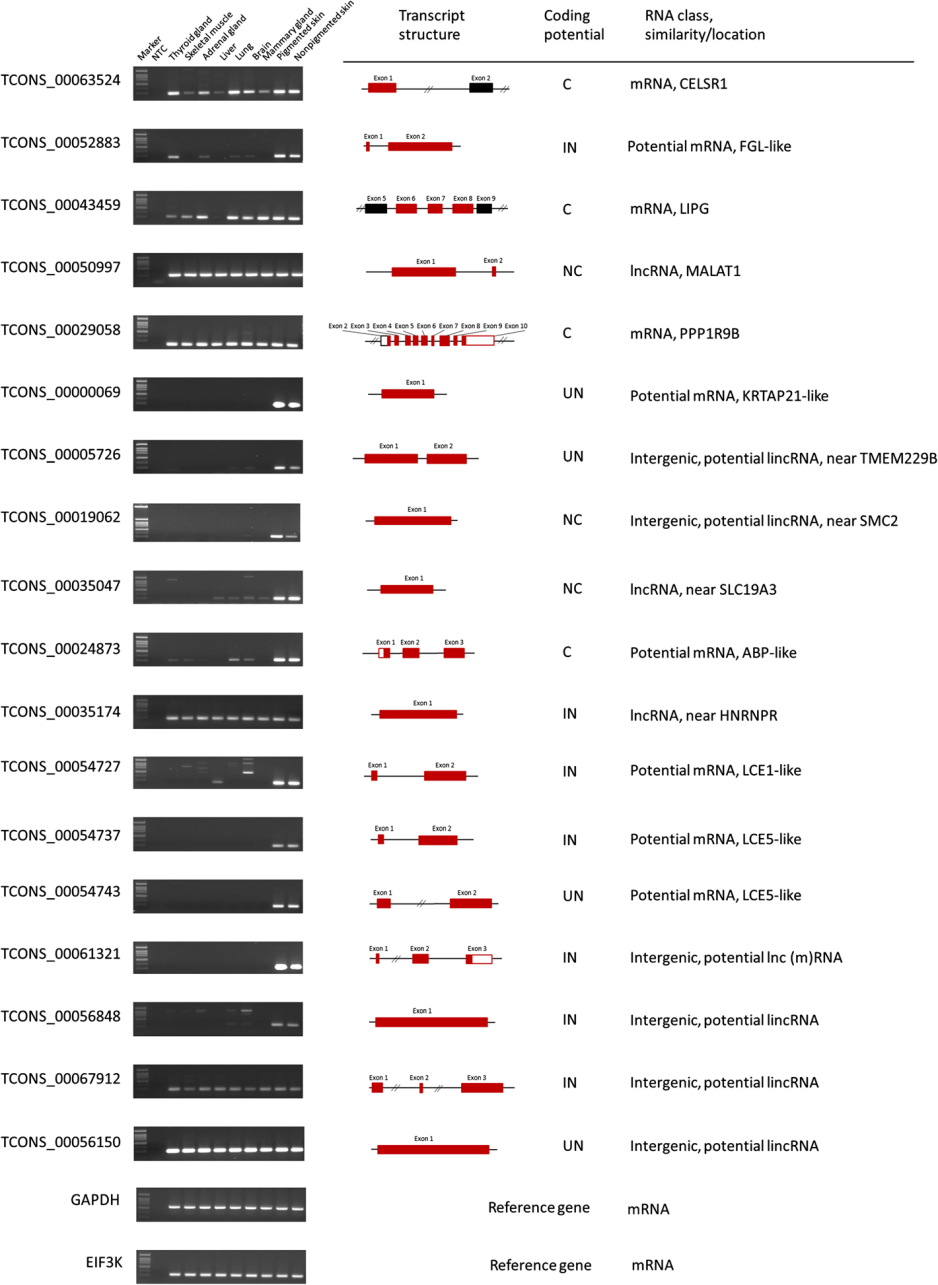
**Tissue-specific expression pattern of selected unknown transcripts.** Transcript structure is illustrated schematically: black boxes represent annotated exons (black framed: *in silico* predicted exons), red boxes indicate novel exonic transcript information (red framed box: untranslated exonic region) obtained in our study. Bioinformatic coding potential prediction by CPC and CPAT tools [55,56], C: protein coding predicted by both tools, NC: noncoding IN: inconsistent prediction between both tools, UN prediction not unambiguous by both tools.

The analysis of our UTs dataset revealed that sequence alignments in diverse RNA databases followed by manual curation can help to refine the bovine genome assembly. Subsequent to previous analyses, experimental validation of 18 selected UTs from different coding potential prediction categories was performed to verify their structure and tissue expression. RT-PCR amplification and subsequent sequencing of the respective amplified cDNA fragments supported the structure and expression pattern of all 18 selected loci transcribed in bovine pigmented and nonpigmented skin. RNA expression profiling in a panel comprising seven different bovine tissues in addition to skin showed that five out of the 18 loci are only or predominantly expressed in skin tissue (Figure 6). Out of the transcripts selected for structure and tissue expression validation a subset of five novel bovine loci will be described in more detail in the following section.

#### XLOC_014395 (PPP1R9B)

The locus XLOC_014395 (with TCONS_00029058 as the longest transcript) represents a novel protein coding gene on BTA19 not previously annotated in the bovine genome assembly. High sequence similarity of the XLOC_014395 locus was found to the human *PPP1R9B* gene (protein phosphatase 1 regulatory subunit 9B neurabin 2, NM_032595) on HSA17. Functionally, PPP1R9B is one of the regulatory subunits of phosphatase-1a and is proposed to be a new tumour suppressor [69]. The presence of several protein orthologs in other species provides strong support for the predicted protein coding potential of the three different structural splice variants of the bovine locus XLOC_014395 supported by RNAseq data (TCONS_00029058, TCONS_00029059 and TCONS_00029060). The CPC score for alternative splice variants ranged from 7.6 to 11.8, whereas the CPAT prediction score was 1. A bovine gene model (gene.326094) had been predicted by GNOMON, but this model overlaps with the adjacent *SAMD14* gene on BTA19 in the bovine reference genome assembly. This predicted annotation on BTA19 is in contrast to our experimental data and the genome organisation in the respective syntenic human chromosome region annotating two separate genes *PPP1R9B* and *SAMD14*. Bovine *PPP1R9B* mRNA is expressed in all bovine tissues analysed including pigmented and nonpigmented skin (Figure 6).

#### TCONS_00024873

The transcript TCONS_00024873 consists of three exons and is a putative novel gene that revealed no similarity to known orthologous transcripts but displayed a divergent expression pattern between pigmented and nonpigmented skin (FPKM: 131.3 vs. 89.0). The transcript was mapped to BTA18 in a poorly annotated region between LOC100847411 and LOC100298523. Both coding potential prediction tools assigned a weak protein coding potential (CPC-S: 1.22, CPAT-S: 0.53) to this transcript. ORF Finder predicted a polypeptide consisting of 117 amino acids including an ATG start codon as well as 3′ and 5′ UTRs. Screening the NCBI protein database found high sequence similarity (99%) to a predicted ovine androgen binding protein (ABP) homolog (LOC101121115, XP_004015679) containing a conserved allergen Feld-I_B domain (pfam09252) and belonging to the secretoglobin superfamily (cd00633) according to the Conserved domain (CDD) database [70]. Androgen, its receptor and binding proteins are known to affect several functions of human skin such as sebaceous gland growth, differentiation and growth of hair, epidermal barrier homeostasis and wound healing, and may play important roles in several skin-related disorders [71,72]. Structure and expression of the bovine androgen binding protein-like transcript TCONS_00024873 were confirmed by RT-PCR. The mRNA expression level of the gene was high in pigmented and nonpigmented skin, moderate in brain and lung and detectable in thyroid gland skeletal muscle (Figure 6).

#### XLOC_025224 (MALAT1)

A prominent noncoding locus (XLOC_025224) highly conserved across species is illustrated by the clustered alignment of several skin transcripts TCONS_00050997, TCONS_00050996, TCONS_00050998 and TCONS_00051000 (5925–6714 bp) on BTA29. This chromosomal region is syntenic to a region on HSA11 where the lncRNA *MALAT1* (metastasis associated lung adenocarcinoma transcript 1 or LINC00047) is located. The bovine transcripts revealed substantial similarity to the human orthologous locus *MALAT1* (NR_002819, 68-82% identity). Hitherto a similar bovine locus was predicted by bioinformatic tools and supported by a variety of ESTs. Two overlapping gene models (gene.1686274 and gene.1684274) had been predicted in the relevant region of BTA29. The bovine *MALAT1* locus was experimentally confirmed by our RNAseq data and RT-PCR experiments. The respective bovine transcripts were concordantly suggested to be ncRNA by both prediction tools (CPC-S: -1.06, CPAT-S: 0.01). Bovine *MALAT1* transcript showed high abundance in pigmented and nonpigmented skin but also in the other tissues included in our bovine tissue panel (Figure 6). MALAT1 was found to be associated with diverse cancer types and to have a function in normal physiology. Tripathi et al. [73] reported that it regulates alternative splicing by interacting with SR (serine/arginine-rich family of nuclear phosphoproteins) splicing factors highlighting a functional role in the regulation of gene expression.

#### TCONS_00035174

Another example for an lncRNA conserved between species is the intronless transcript TCONS_00035174 (5726 bp) that was concordantly predicted as putative noncoding (CPC-S: -0.15, CPAT-S: 0). It was mapped on BTA2 and revealed high similarity to the human lincRNA n337771 (also designated as lnc-HNRNPR-1:1). The mapping position on BTA2 adjacent to the bovine *HNRNPR* gene highlighted a syntenic region on HSA1 near the human *HNRNPR* gene. The respective bovine transcript displayed moderate expression in all tissues investigated including pigmented and nonpigmented skin (Figure 6).

#### TCONS_00061321

In a recent RNAseq study in sheep skin [74], two novel presumably noncoding transcripts were found to be differentially expressed between black and white skin but did not display any similarity to sequences in the NCBI database including ESTs. This result underlines specific and restricted expression patterns of lncRNAs and also illustrates the limited current knowledge about ruminant ncRNAs. We also retrieved a variety of UTs in bovine skin showing no sequence similarity to annotated loci of other species. This case is exemplified by the transcript TCONS_00061321 that was mapped on BTA5 (between *USP44* and *GLYCAM1*). This locus revealed no sequence similarity to human and mouse transcripts. It consists of three exons and displays alternative splice variants. One of the exons showed identity to an exon of a bovine locus (gene.473414) predicted by GNOMON. Comparative sequence analysis across other mammalian species showed that sequence similarity of TCONS_00061321 was detected for two exons mapping in a syntenic chromosome region on ovine chromosome 3 (89-94%) between *USP44* and *GLYCAM1*. Furthermore, a high sequence similarity was observed with several ovine ESTs from cDNA libraries prepared from adult ovine skin (e.g., CF115983) or wool follicles in different phases of hair growth cycle (e.g., EE847431, EE857040) as well as with a transcript from an RNAseq transcriptome analysis of goat skin in the anagen phase of hair growth cycle (KA343470). The coding capacity of the bovine transcript TCONS_00061321 was not consistently predicted by the two coding potential prediction tools (CPC-S: -0.31 and CPAT-S: 0). However ORF Finder predicted a polypeptide consisting of 110 amino acids but without an ATG start codon. Screening the NCBI protein database with this predicted amino acid sequence did not find any sequence similarity to known proteins. However, searching for conserved domains using the CDD tool [70] identified a transposase zinc-binding domain which is found to be located at the N-terminus of transposases belonging to the IS91 family (pfam14319). RNA expression profiling across bovine tissues revealed that the transcript TCONS_00061321 is only expressed in skin (Figure 6). We postulate that this transcript is a skin-specific transcript in ruminants. This hypothesis is supported by the existence of ESTs in wool and skin from sheep whereas transcript databases of other mammalian species (human, mouse, pig, horse and dog) revealed no similar transcript sequences.

## Conclusions

In this study we focused on transcripts that were discovered by deep transcriptome sequencing and were not yet annotated in the current bovine genome assembly. As a result, we generated the first catalogue of potential lncRNAs for bovine skin based on a whole transcriptome RNAseq approach. Out of 10,884 unknown transcripts we predicted 4,849 putative lncRNAs, mapped them on the bovine reference genome assembly and characterised their positions compared to adjacent annotated loci. Furthermore, we were able to detect novel bovine genes and to refine known transcript loci, the structure of which was not completely annotated in the bovine reference genome assembly. Importantly, the expression of a number of selected novel or refined transcripts including putative lncRNAs was verified experimentally in bovine skin and in several bovine tissues. Collectively, the results presented here reveal that the range, depth and complexity of the bovine transcriptome are far from being fully characterised.

The results also suggest that unknown, not annotated transcripts yielded from whole transcriptome sequencing appear to harbour an as yet unexplored reservoir of novel functional RNAs. As such they should not be ignored in surveys of functional transcripts or other transcriptomic and genomic studies. However, it is still difficult to annotate unknown RNA unequivocally as protein coding or noncoding exclusively based on available bioinformatic prediction tools. Manual meticulous curation of primary prediction results, careful interpretation of data and molecular experimental validation are critical to evaluate the presence and functional role of ncRNAs in a transcriptome.

Prospectively, the identification and molecular understanding of the pigmentary and epidermal systems in mammals like cattle should contribute information about pigmentation processes and disorders. This is exemplified by genes, which were reported to cause fancy coat colour variation in mouse and were often associated with serious human disorders, e.g., in neural function, sight, hearing or blood clotting [23] indicating the value of comparative genome data.

## Competing interests

The authors declare that they have no competing interests.

## Author’s contributions

CK and RW performed the RNAseq experiment. CK conceived the study, performed transcript mapping and assembly and critically revised the manuscript. CK and FH carried out bioinformatic data analyses. RW investigated unknown transcripts, performed manual data curation, validation experiments and analyses and wrote the manuscript. All authors read and approved the final manuscript.

## Supplementary Material

Additional file 1 **Primer sequences used for RT-PCR.** T: annealing temperature of primers in the PCR assay, region: refers to the transcript structure. Application: The respective primer pair was applied for validation of transcript structure or/and expression analysis using RT-PCR. Click here for file

Additional file 2 **Unknown bovine skin transcripts predicted to possess protein coding potential.** Position: mapping position on the bovine genome assembly (UMD3.1); nexon: number of exons; cpat cpc: scores for coding potential [55,56]; nearest_neighbour: nearest neighbouring locus; distance_neighbour: distance of the respective transcript to the nearest neighbouring gene; Blast_human Blast_bovine: sequence similarity to human or bovine genomes identified by BLAST search; pig_FPKM nonpig_FPKM: fragments per kb per transcript per million mapped reads in pigmented and nonpigmented skin. Click here for file

Additional file 3 **Unknown bovine skin transcripts predicted to possess protein noncoding potential.** Position: mapping position on the bovine genome assembly (UMD3.1); nexon: number of exons; cpat, cpc: scores for coding potential [55,56]; nearest_neighbour: nearest neighbouring locus; distance_neighbour: distance of the respective transcript to the nearest neighbouring gene. Click here for file

Additional file 4 **Sequence similarity of unknown transcripts conserved between species.** Similarity_human similarity_bovine: similarity to human or bovine genomes identified by BLAST search; lncipedia, gencode, ncode: similarity to known ncRNAs found by screening the ncRNA databases LNCipedia [51], Gencode [9,50], Noncode [52]. Click here for file
