# Supplementary material for: Identification of novel transcripts and noncoding RNAs in bovine skin by deep next generation sequencing
Source: BMC Genomics. 2013 Nov 14;14:789. doi: 10.1186/1471-2164-14-789 (PMC3833843; doi:10.1186/1471-2164-14-789)
Supplement: Additional file 1 — Primer sequences used for RT-PCR. T: annealing temperature of primers in the PCR assay, region: refers to the transcript structure. Application: The respective primer pair was applied for validation of transcript structure or/and expression analysis using RT-PCR. [file 1471-2164-14-789-S1.docx]

| **Transcript_ID** | **Primer_ID** | **Sequence (5'-3')** | **Region** | **Amplicon (bp)** | **T (°C)** | **Application** |
| --- | --- | --- | --- | --- | --- | --- |
| TCONS_00043459 | LIPG_F1 | CGT AGA TGC GGT CAA TAA CAC | exon 3 |  |  |  |
|  | LIPG_R1 | TTG CTG TTC CTC TTG TTC CTT G | exon 6 | 606 | 62 | structure |
|  | LIPG_F2 | CGG GCT GTG GAC TCA ACG ATG | exon 1/2 |  |  |  |
|  | LIPG_R2 | TTG AAG CGG TTG GAG TCT GTG | exon 2 | 163 | 64 | RNA expression |
| TCONS_00029058 | PPPP1R9_F3 | GAC ATG GGC CTT GAG AAG CTG G | exon 3 |  |  |  |
|  | PPPP1R9_R1 | GTT CTC CTC CAC GCT CTG CTC C | exon 8 | 595 | 68 | structure |
|  | PPPP1R9_F1 | GAC TGA GGC CGA GAT CCA GCA G | exon 7 |  |  |  |
|  | PPPP1R9_R1 | GTT CTC CTC CAC GCT CTG CTC C | exon 8 | 109 | 66 | RNA expression |
| TCONS_00063524 | CELSR1_F1 | GGT GGG GTC CGT GGT GGC GAG | exon 1 |  |  |  |
|  | CELSR1_R1 | GGA TGT TGA AGA CGA AGA CGG | exon 2 | 704 | 62 | structure |
|  | CELSR1_F2 | AAG TGT CAG TTT CCG ACG GCA T | exon 1/2 |  |  |  |
|  | CELSR1_R2 | ACA GCA GCG GGG ACA GGA AC | exon 2 | 144 | 64 | RNA expression |
| TCONS_00052883 | FLGL_F1 | AGC AAC ACA AAA GGG GCA CTA C | exon 1 |  |  |  |
|  | FLGL_R1 | TAT CTG TGT CTG TGG CAA ATC C | exon 2 | 138 | 62 | structure, RNA expression |
| TCONS_00067912 | TC67912_F1 | CTG GGG GTC GGC TCG GAA TC | exon 1 |  |  |  |
|  | TC67912_R1 | TCT GGG CTG GGT GGT CAT AAC | exon 3 | 136 | 64 | structure, RNA expression |
| TCONS_00056848 | TC56858_F2 | CAA CCA GGG AAC CGC AAT AAC | exon 1 |  |  |  |
|  | TC56858_R1 | ACT CCC CTG CCC CAA ACC TC | exon 1 | 469 | 66 | structure, RNA expression |
| TCONS_00024873 | TC24873_F1 | CCA GAG AGC TGA CCT TTG AGA TG | exon 1 |  |  |  |
|  | TC24873_R1 | CCT GAC AGA GAC CAT ATT ACG TG | exon 3 | 421 | 66 | structure |
|  | TC24873_F2 | CGC TCA GTT TGG TCA GTG CTA C | exon 2 |  |  |  |
|  | TC24873_R2 | TGA GGA CAG TAC ATA GCC ATT GC | exon 3 | 158 | 66 | RNA expression |
| TCONS_00005726 | TC05726_F1 | TAC TTT TCC TTC CTA TGA CTC TG | exon 1 |  |  |  |
|  | TC05726_R2 | AGA CTG AAG CAT AGA TAG GTT AC | exon 2 | 370 | 62 | structure |
|  | TC05726_F1 | TAC TTT TCC TTC CTA TGA CTC TG | exon 1 |  |  |  |
|  | TC05726_R1 | AGA GTG AGA AAG GGC CTT CAT C | exon 1/2 | 127 | 62 | RNA expression |
| TCONS_00050997 | MALAT1_F | GCT TTT CAG GAT TTT GGC TTT C | exon 1 |  |  |  |
|  | MALAT1_R | ACA AGT AAG CCC CAC CCT CTC C | exon 1 | 159 | 60 | structure, RNA expression |
| TCONS_00050997 | MALAT1_F2 | TTT TAA ATG TGG GGA TTG GGA AC | exon 1 |  |  |  |
|  | MALAT1_R2 | TCA AAA CAG GCT TCT CCA ATC AC | exon 2 | 149 | 62 | structure |
| TCONS_00051000 | MALAT1_F2 | TTT TAA ATG TGG GGA TTG GGA AC | exon 1 |  |  |  |
|  | MALAT1_R3 | GAC ATA GAA AAA ACT TGT TCA CCT G | exon 2 | 128 | 62 | structure (INDEL2-specific) |
| TCONS_00051000 | MALAT1_F4 | AGA ACG AAT TTA ATT TAA GAG GCT | exon 1 |  |  |  |
|  | MALAT1_R4 | CAC CCT CTC CTC CCT TCC CTC | exon 1 | 190 | 60 | structure (INDEL1-specific) |
| TCONS_00035174 | TC35174_F1 | GAT TCC AGG GTG TGT TAT TTA G | exon 1 |  |  |  |
|  | TC35174_R1 | TCC TCC CCT CAA TCT GTT TTA G | exon 1 | 134 | 60 | structure, RNA expression |
| TCONS_00019062 | TC19062_F1 | AGT AAG AAT AAA AAT CAC CAA TCA C | exon 1 |  |  |  |
|  | TC19062_R1 | AGG ATG ATA AAA AAT GAC TTC TGA C | exon 1 | 184 | 62 | structure, RNA expression |
| TCONS_00056150 | TC56150_F1 | TAC TGA ATC CCA AGA GCC ATA G | exon 1 |  |  |  |
|  | TC56150_R1 | TGC TGA GTC TTT GTT GCC GTG | exon 1 | 148 | 62 | structure, RNA expression |
| TCONS_00035047 | TC35047_F1 | TTG GAC CCT GAG AAG TTT ATT G | exon 1 |  |  |  |
|  | TC35047_R1 | TCA TCC AGA CCA GAA TCC CCA C | exon 1 | 146 | 60 | structure, RNA expression |
| TCONS_00054743 | TC54743_F1 | CAG TGT TGA ACC AGC TCC ATT C | exon 1 |  |  |  |
|  | TC54743_R2 | CCG TTC TCT TCT TCC AAT CTT C | exon 2 | 469 | 62 | structure |
|  | TC54743_F1 | CAG TGT TGA ACC AGC TCC ATT C | exon 1 |  |  |  |
|  | TC54743_R1 | GGG CAC TTG GGA GGA CAC TTA G | exon 2 | 133 | 64 | RNA expression |
| TCONS_00000069 | TC00069_F2 | TAA AGG TCC ATG GGG AGA AGA C | exon 1 |  |  |  |
|  | TC00069_R1 | TCA GAT TTC AAG GAC ATC GCT C | exon 1 | 478 | 64 | structure |
|  | TC00069_F1 | GCT GCT AGA ACA TCA CCC TTA G | exon 1 |  |  |  |
|  | TC00069_R1 | TCA GAT TTC AAG GAC ATC GCT C | exon 1 | 161 | 62 | RNA expression |
| TCONS_00061321 | TC61321_F2 | TTC CTG CTT CTT CTC CTG CCT C | exon 1 |  |  |  |
|  | TC61321_R1 | AAG TAG TCG CAG TGA AAC AAA TC | exon 3 | 413 | 58 | structure |
|  | TC61321_F1 | TGA TGA CTG CCT CTT TTA TTT TC | exon 2 |  |  |  |
|  | TC61321_R1 | AAG TAG TCG CAG TGA AAC AAA TC | exon 3 | 154 | 58 | RNA expression |
| TCONS_00054737 | TC54737_F1 | CTC CAC TGA CCA GGT TTA CTG AC | exon 1/2 |  |  |  |
|  | TC54737_R2 | GGG CAC AGA GGG AGA GGA TAG | exon 2 | 474 | 66 | structure |
|  | TC54737_F1 | CTC CAC TGA CCA GGT TTA CTG AC | exon 1/2 |  |  |  |
|  | TC54737_R1 | ACT TGG GGG GAC ACT TGG CAG | exon 2 | 135 | 66 | RNA expression |
| TCONS_00054727 | TC54727_F1 | CGT GTG CGC TGA TCC TGA AC | exon 1/2 |  |  |  |
|  | TC54727_R2 | GGA TTG GCA GAT GAG TTT TTA GG | exon 2 | 518 | 62 | structure |
|  | TC54727_F1 | CGT GTG CGC TGA TCC TGA AC | exon 1/2 |  |  |  |
|  | TC54727_R1 | GGA GAC TGG GGG ACA CTT TGG | exon 2 | 131 | 62 | RNA expression |
